# An Improved YOLOv8n Used for Fish Detection in Natural Water Environments

**DOI:** 10.3390/ani14142022

**Published:** 2024-07-09

**Authors:** Zehao Zhang, Yi Qu, Tan Wang, Yuan Rao, Dan Jiang, Shaowen Li, Yating Wang

**Affiliations:** 1 School of Information and Artificial Intelligence, Anhui Agricultural University, Hefei 230036, China; 23723186@stu.ahau.edu.cn (Z.Z.);; 2 Key Laboratory of Agricultural Sensors, Ministry of Agriculture and Rural Affairs, Hefei 230036, China; 3 Anhui Provincial Key Laboratory of Smart Agricultural Technology and Equipment, Hefei 230036, China; 4 College of Engineering, Anhui Agricultural University, Hefei 230036, China

**Keywords:** fishery resource investigation, fish detection, computer vision, YOLOv8n

## Abstract

**Simple Summary:**

Underwater fish species are an important direction in fishery resource surveys. Rapidly determining species of underwater fish can improve the efficiency of fishery resource surveys. Therefore, this study proposes an effective method for underwater fish measurement, which can quickly acquire underwater fish species. The experimental results demonstrate the accuracy and superiority of our method. The proposed method improves the efficiency of fishery resource surveys and provides crucial data support for the precise management of fishery resources.

**Abstract:**

To improve detection efficiency and reduce cost consumption in fishery surveys, target detection methods based on computer vision have become a new method for fishery resource surveys. However, the specialty and complexity of underwater photography result in low detection accuracy, limiting its use in fishery resource surveys. To solve these problems, this study proposed an accurate method named BSSFISH-YOLOv8 for fish detection in natural underwater environments. First, replacing the original convolutional module with the SPD-Conv module allows the model to lose less fine-grained information. Next, the backbone network is supplemented with a dynamic sparse attention technique, BiFormer, which enhances the model’s attention to crucial information in the input features while also optimizing detection efficiency. Finally, adding a 160 × 160 small target detection layer (STDL) improves sensitivity for smaller targets. The model scored 88.3% and 58.3% in the two indicators of mAP@50 and mAP@50:95, respectively, which is 2.0% and 3.3% higher than the YOLOv8n model. The results of this research can be applied to fishery resource surveys, reducing measurement costs, improving detection efficiency, and bringing environmental and economic benefits.

## 1. Introduction

Fishery resources are one of the most important resources for human survival and possess extremely high value in improving quality of human life [1]. With the growth in population and the advancement of fishery technology, the issues of overexploitation of fishery resources and environmental pollution have become increasingly prominent, threatening not only ecological balance but also the long-term development of fishery resources. Therefore, it is necessary to conduct regular and fair assessments of fishery resources to effectively manage and protect them [2]. Regular resource assessments are crucial for the scientific management of fishery resources [3]. Investigating fishery resources in marine and freshwater environments, including species, quantity, distribution of fish, and related ecological and environmental information, can provide a scientific basis for formulating fishing quotas, endangered species protection, and environmental protection measures. Moreover, research on fishery resources can be used to monitor and understand changes in the ecological environment, which is crucial for preventing and managing the overexploitation of fishery resources.

The use of computer vision technologies is especially important in this situation. Conventional techniques for surveying fisheries’ resources are frequently expensive, labor-intensive, and ineffective. Automated and exact surveys of fisheries’ resources can benefit greatly from computer vision technology’s ability to rapidly and reliably identify and monitor underwater objects, such as fish species, numbers, and behavioral patterns [4]. By thoroughly analyzing fish behavior and migration patterns, this technology not only greatly reduces the need for human resources and increases the effectiveness of investigations, but it also offers a strong scientific foundation for the development of fishery protection policies and sustainable resource use.

The application of target detection technology in fishery resource surveys has significant prospects. By analyzing images using pre-trained deep learning algorithm models, fish are automatically identified and classified, and accurate target detection is performed based on their morphological characteristics and color. This technology improves the efficiency and accuracy of fishery resource surveys and overcomes the shortcomings of traditional methods that are time-consuming and have a greater impact on the environment. There has been research on the use of target detection algorithms to identify underwater fish. However, the accuracy of underwater target recognition is significantly impacted by the specificity and complexity of underwater imaging [5]. Small fish, in particular, tend to be relatively small in images, which might cause issues with missed or incorrect identification by traditional target detection algorithms. Furthermore, the underwater environment’s light and water quality frequently fluctuate, which makes fish’s visual features hazy, warped, or darker. Moreover, fish may group themselves or become hidden by nearby items. Therefore, target recognition becomes more challenging in the aquatic environment. Algorithms with strong identification and generalization skills are needed to tackle these issues in detection and recognition. As a result, this study suggests a new model, BSSFISH-YOLOv8, and modifies and optimizes these difficulties using the minimum volume model YOLOv8n in YOLOv8 as the foundation. The following are the primary contributions of the study:

(1) The original YOLOv8n convolution module was replaced with the SPD-Conv module due to the low resolution of captured images and low clarity of the underwater environment;

(2) To deal with the problem of missing detection and false detection caused by underwater organisms accumulating around and blocking each other, the dynamic sparse attention mechanism BiFormer was introduced in the backbone network;

(3) A shallow layer with a smaller receptive field was chosen for improvement to improve YOLOv8n’s ability to detect small fish targets in complex environments. The network’s sensitivity to small target identification can be effectively increased by varying the feature fusion mode and raising the feature detection scale.

## 2. Related Works

Traditional fishery resource survey methods mainly rely on two basic types of techniques, direct and indirect [6]. Direct methods involve catching or observing aquatic life, whereas indirect methods use a variety of tools and techniques to estimate fishery resources. Common direct methods include trawl surveys and electrofishing, which are commonly used to assess fish populations and biomass. Trawl surveys capture fish by dragging a net over a prescribed path and time, and electrofishing uses an electric current to briefly paralyze fish and bring them to the surface for capture. The study by Macnaughton et al. [7] focused on electrofishing technology, which is studied by using an electric current to temporarily immobilize fish. It is particularly effective in streams and shallow water ecosystems and is mainly used for fish population assessment. Another direct method is visual surveys, which are methods of directly observing marine life, primarily through divers or underwater camera equipment. For example, Jordan et al. [8] estimated the abundance of endangered benthic stream fish by directly observing fish through diving or underwater cameras. This method intuitively provides information on fish behavior, abundance, and habitat by utilizing high-resolution data. Indirect methods include acoustic surveys and environmental DNA(eDNA) techniques, among others. Acoustic surveys use sonar technology to estimate the density and distribution of fish schools. This method works by emitting sound waves and catching the sound waves reflected by fish or other marine life. It can quickly cover large areas of the sea and is suitable for large-scale fish monitoring [9]. For example, Bollinger et al. [10] used side-scan sonar echoes to collect data about fish and calibrated sonar readings to obtain accurate fish counts. Environmental DNA technology involves extracting and analyzing DNA fragments from water samples to identify species present in a specific area. Environmental DNA sampling can cover a large geographical range and creates minimal interference with organisms [11]. Doi et al. [12] used environmental DNA technology to analyze the genetic material in water and provided a broad overview of biological diversity. This study demonstrates the importance of environmental DNA technology in estimating fish abundance, biomass, and spatial distribution in river ecosystems.

In recent years, computer information technology has developed rapidly, and computer vision has made significant progress [13]. Moreover, target detection technology has become an important tool in the field of underwater biological monitoring [14,15], so more and more target detection methods are used in the field of fishery resource investigation.

Traditional target detection algorithms are mainly based on a sliding window mechanism, where the image is covered by multiple windows of different sizes to select areas that may contain targets. Feature extraction is crucial in traditional methods, including scale-invariant feature transform (SIFT) [16], histogram of oriented gradients (HOG) [17], deformable part models (DPMs) [18], local binary patterns (LBPs) [19], and other manual feature extraction methods. After feature extraction, traditional methods usually use machine learning classifiers, such as support vector machine (SVM) [20] and adaptive boosting (Adaboost) [21], to classify whether the extracted features belong to a specific target category. These classifiers require large amounts of labeled data when training and may need to be retrained and tuned for different tasks. Hsiao et al. [22] proposed a local ranking method using sparse representation classification to detect fish, using multiple bounding boxes to distinguish fish from other objects. Although this method has high accuracy, it lacks real-time performance. On the other hand, Cutter et al. [23] adopted a cascade classifier to detect underwater fish by creating Haar-like features for underwater images for classification. This method is specifically designed for underwater images and has good results in specific scenarios. However, traditional fish detection technology usually requires manual screening of suitable features and has inconsistent results in different underwater environments, because the robustness of traditional technology is relatively weak. To improve the adaptability and performance of detection algorithms, some scholars use fish detection methods based on deep learning.

In recent years, the field of underwater fish detection has made significant progress with the application of deep learning technology. Due to poor underwater image quality and unconstrained fish movements, traditional hand-designed feature extraction methods or target detection algorithms based on convolutional neural networks cannot meet the detection requirements of real underwater scenes. Li et al. [24] used Fast R-CNN to detect and identify fish species in underwater images, achieving an average accuracy of 81.4% on a complex underwater environmental dataset with 12 fish species. Zhao et al. [25] proposed the Composited FishNet model. This is a composite fish detection framework that enhances feature information output and improves the utilization of this information. This novel method designs a composite backbone network, CBresnet, by improving the residual network to learn the changing information of underwater scenes and designs an enhanced path aggregation network, EPANet, to better integrate network output and achieve complex underwater environment identification and location. Wageeh et al. [26] proposed a target detection algorithm to enhance the clarity of blurred water images. The algorithm contains two main steps, namely, using the multi-scale Retinex (MSR) [27] algorithm to enhance the image or video to improve clarity and using the YOLOv3 target detection algorithm to detect objects in the image. However, as an image enhancement algorithm based on physical models, the MSR algorithm has a relatively slow processing speed, which may limit the application scope of the algorithm, especially in application scenarios where real-time performance needs to be considered. The YOLO-Fish [28] model is one of the most famous models in this field, and it is specially designed to detect fish in realistic underwater environments. The model uses sophisticated algorithms to address the challenges posed by underwater conditions, proposing a robust method of fish detection. The focus of YOLO-Fish is to improve the accuracy of the model in identifying fish species, especially in poor visibility conditions. In addition, the research by Wang et al. [29] introduced a diseased fish detection model tailored for intensive aquaculture. The researchers improved the YOLOv5 network, emphasizing the importance of accurate fish detection models in aquaculture. The study by Patro et al. [30] used the YOLOv5-CNN detection model for underwater fish detection, emphasizing the advantages of this model over other methods in terms of speed and accuracy. However, this method requires a large number of training data to improve the accuracy and reliability of the algorithm, which also brings great limitations to its practical application. The recent availability of YOLOv8 is a further enhancement to the performance and flexibility of target detection. YOLOv8 has demonstrated higher performance on public datasets such as COCO2017. To solve the problem of dense underwater fish populations and plant occlusion, Li et al. [31] designed a model algorithm based on YOLOv8, which achieves better detection by adding an RT-DETR module. Aiming at the difficulty of accurate identification caused by small underwater target fish occupying small image spaces with fast swimming speeds, Qin et al. [32] introduced deformable convolution (AKConv) into YOLOv8, which can automatically adjust the convolution kernel size according to the size and shape of objects and capture the shape and contour features of fish more accurately and quickly. These show the strong application potential of YOLOv8.

However, existing technologies focus on the detection of general scenes and ignore the dynamic recognition of fish. Especially when detecting fish in natural waters, factors such as occlusion and low definition greatly affect the recognition efficiency and accuracy [33]. At the same time, the inefficiency and ambiguity of image feature information faced by small target individuals are still major problems in the field of target detection [34,35]. Current detection technology has limited capabilities in solving fish occlusion, low definition, and small target problems and has not yet met the needs of fishery research. Improvements in target detection technology are critical to solve these problems, pushing the field toward more efficient and accurate underwater fish detection and identification.

## 3. Materials and Methods

### 3.1. YOLOv8 Detection Algorithm

**Figure 1:**
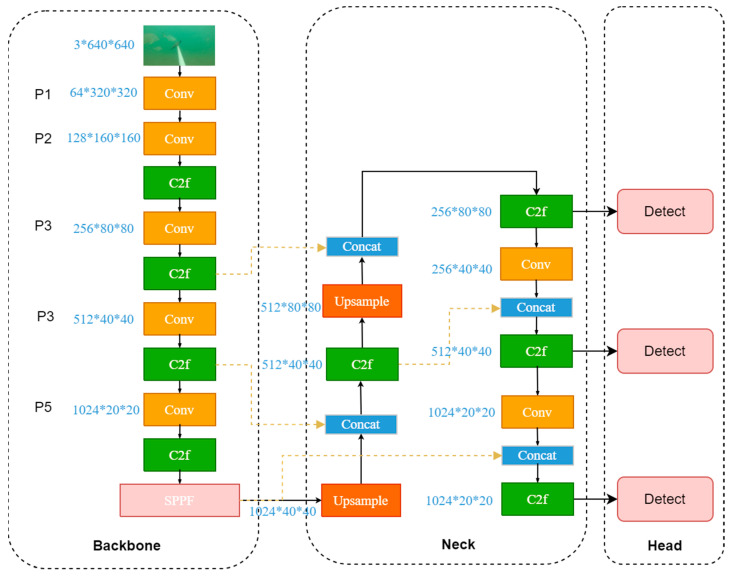
YOLOv8 network structure.

YOLOv8 has been significantly improved based on YOLOv5 by introducing a more complex network structure. It combines numerous residual units with multiple branches, thereby outperforming YOLOv5 in terms of speed and accuracy. Compared with YOLOv5, the main difference of YOLOv8 is that the C3 module of the backbone layer is replaced by the C2f module. This new module introduces a gradient shunt connection to enhance the information flow within the feature extraction network while still maintaining a lightweight design. In PAN-FPN, the convolution operation after upsampling in YOLOv5 is eliminated, achieving a more streamlined configuration. In sample matching, a Task-Aligned Assigner is used for matching. Compared with previous excellent algorithms in the YOLO series, YOLOv8 is an advanced and cutting-edge algorithm with prominent detection accuracy and speed. The network architecture of YOLOv8 is mainly divided into three parts: a backbone, neck, and head. The overall structure is shown in Figure 1.

### 3.2. The Proposed BISSFISH-YOLOv8

#### 3.2.1. SPD-Conv Module

The SPD-Conv module is a novel CNN building block proposed by Sunkara et al. [36]. It completely moves away from the stepwise convolution and max pooling used in previous models. It is used to improve detection performance on low-resolution images and small objects and can be integrated into most CNN architectures. It implements a convolutional network composed of an SPD layer and a Conv layer, where the SPD layer converts the spatial dimension to the depth dimension, and the Conv layer implements non-strided convolution. The space-to-depth (SPD) layer mainly converts the spatial dimension of the input feature map into the channel dimension, thereby reducing the spatial size while maintaining the integrity of the channel information. This transformation is achieved by mapping each pixel or feature to a different channel, resulting in a reduction in the spatial dimension and an increase in the channel dimension. Following the SPD layer is the non-strided convolution layer, a standard convolution operation. Unlike strided convolution, non-strided convolution does not move on the feature map but performs independent convolution on each pixel or feature, which helps to alleviate the over-subsampling problem that may be caused by the SPD layer while maintaining more detailed information. This combination method effectively reduces the size of the spatial dimension while ensuring the integrity of channel information, thereby improving the performance of low-resolution images and small object detection.

**Figure 2:**
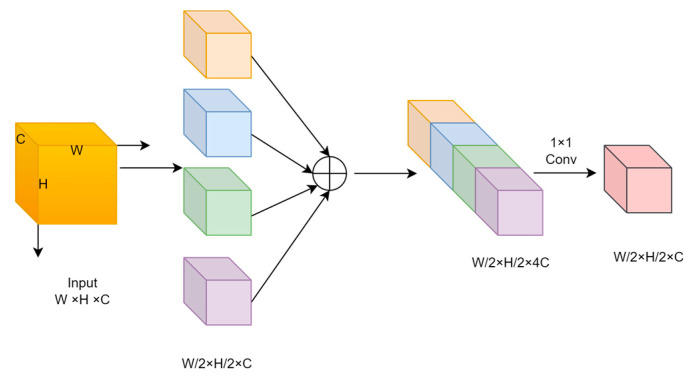
SPD-Conv module structure.

The specific structure of the SPD-Conv module is shown in Figure 2. Firstly, the input feature map is of size W × H × C. After interval sampling, four feature maps are obtained, X*_1_*, X*_2_*, X*_3_*, and X*_4_*_,_ respectively, whose dimensions are W/2 × H/2 × C. Subsequently, the four feature maps are spliced to obtain the feature map X*_cat_*, whose dimensions are W/2 × H/2 × 4C, as in Formula (1):

X*_cat_* = [X*_1_*; X*_2_*; X*_3_*; X*_4_*] (1)

After that, the feature map X_1×1_ is obtained by 1 × 1 convolution, whose dimensions are W/2 × H/2 × C, as in Formula (2):

X*_1__×1_* = Conv*_1__×1_* (X*_cat_*) (2)

Finally, feature extraction is performed by 3 × 3 convolution, as in Formula (3):

X_*out*_ = Conv_*3×3*_(X_*1×1*_) (3)

The overall formula can be expressed as in Formula (4):

X*_out_* = Conv*_3__×3_*(Conv*_1__×1_*([X*_1_*;X*_2_*;X*_3_*;X*_4_*])) (4)

Through this process, the SPD-Conv module halves the spatial dimension without changing the number of channels, enhancing the sensory field and expression of features.

To solve the problem of low clarity in the underwater environment and low resolution of captured images, this study chose to use the SPD-Conv module to replace the original convolution module of YOLOv8n. 

#### 3.2.2. BiFormer Attention

Attention mechanisms [37] are a technology that is commonly used in many fields such as computer vision and natural language processing. They mimic the way humans allocate attention, allowing neural networks to focus on key or relevant parts of input information. The sparse attention mechanism has been popularized in the field of target detection [38]. As a special form of attention mechanism, sparse attention limits the scope of attention and significantly reduces computational complexity by adopting local window and position encoding strategies. This mechanism shows better scalability in tasks such as image recognition, target detection, and image segmentation. A noted problem is that feature fusion is not perfect, because the fused features will lose some important detailed information and affect detection accuracy [39]. The introduction of the attention mechanism can effectively solve this problem. It evaluates the similarity or correlation between different features and assigns appropriate weights to ensure that the network focuses on features that contain key information. In addition, combining the attention mechanism and feature fusion network can effectively improve the common missed detection and false detection problems in underwater biological detection. 

**Figure 3:**
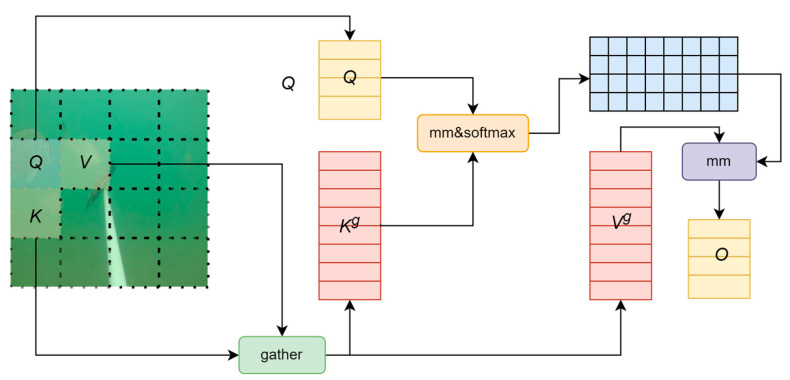
BiFormer attention structure.

The structure of Biformer is shown in Figure 3. BiFormer is developed based on the Transformer model, which adds a dynamic attention mechanism to the standard Transformer model. This mechanism is called Bi-level Routing Attention (BRA) and is a form of dynamic sparse attention. The BiFormer attention mechanism aims to achieve more flexible calculation allocation and deep understanding of content, thereby giving the model dynamic query-aware sparsity. This mechanism better preserves fine-grained details by being based on sparse sampling. Since this module uses sparse sampling rather than downsampling, it can retain information details more carefully and completely [40]. The introduction of BiFormer effectively alleviates the problem of loss of feature details. This method optimizes the fusion process between features by focusing on key features in the image, ensuring that the network can effectively distinguish and identify overlapping or partially occluded fish individuals. Integrating the BiFormer attention mechanism into the feature fusion process allows the network to pay more attention to feature information related to individual fish at multiple scales while maintaining the light weight of the model and avoiding excessive computational burden.

The process of BiFormer can be divided into three steps:

First, a feature map is input, the image is divided into multiple coarse-grained regions, and the most relevant query Q, key K, and value tensor V are obtained at the coarse-grained region level through linear mapping to obtain partial routing regions to focus on only these small amounts of key areas.

Then, the attention mechanism is applied to the fine-grained tokens (K^g^, V^g^) in these selected routing areas, and the most relevant key-value pairs are selected to participate in subsequent calculations by analyzing the similarity between key K and query Q. This is shown in Formulas (5) and (6). Among them, K^g^ and V^g^ are the collected key-value tensors, I^r^ represents the index of the i-th row including the top K most relevant regions in the i-th region, and K refers to obtaining the key-value pairs in the first K relevant windows.

K^g^ = gather (K, I^r^) (5)

V^g^ = gather (V, I^r^) (6)

Finally, attention processing is performed on the collected K^g^ and V^g^ tensors, and the local context enhancement term LCE(V) is added to obtain the output tensor O, thereby applying a fine-grained token-to-token attention mechanism between different regions. Its calculation formula is as shown in Formula (7):

O = Attention(Q, K^g^, V^g^) + LCE(V) (7)

This study chose to integrate the BiFormer sparse attention mechanism into the improved feature fusion part. This mechanism can automatically adjust the attention weight according to the characteristics of the input image, giving different levels of attention to different positions and features, to focus more on underwater targets. Feature information is obtained while maintaining the lightweight and computational efficiency of the model.

#### 3.2.3. Small Target Detection Layer (STDL)

**Figure 4:**
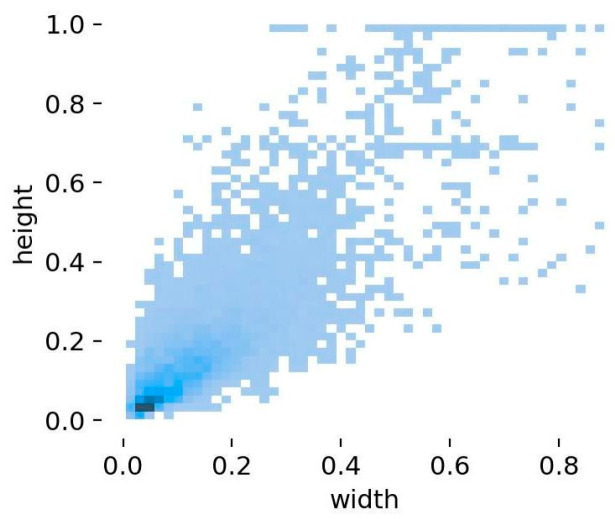
Target size distribution of the dataset (Darker colours represent a greater number of distributions).

The definition of small targets generally covers two aspects. Absolutely small targets refer to targets whose target pixels in the dataset are less than 32 × 32, and relatively small targets refer to targets whose target size accounts for less than 10% of the image size [41]. In the dataset used in this article, the size of the objects is unevenly distributed. By observing the ratio distribution of targets in the dataset, we can find that the ratios of a large number of targets fall between 0 and 0.2, as shown in Figure 4. This shows that small targets occupy a larger proportion of the dataset. Due to the large number of small targets, accurate detection of these small targets will become more difficult.

In the target detection process, the detection accuracy of small targets is often very low compared with normal targets. In the entire image, the proportion of small target pixels is relatively low, and the model learns very little background information. In addition, small targets are often accompanied by other large targets, and these large targets often dominate the learning process during the detection process, making small targets difficult to detect. In this case, the reliability of the convolutional neural network decreases significantly. In the early layers of the convolutional neural network structure, the image resolution is suitable for studying large targets, and a large amount of redundant information can be filtered well in the stride convolution, making the features learned by the model better. However, when the number of image pixels is small, or the detection target is small, the amount of redundant data is smaller. In this case, strided convolution and pooling will lead to the loss of fine-grained information, resulting in insufficient learning of small targets by the algorithm, which is an important reason for the low efficiency of small target detection.

**Figure 5:**
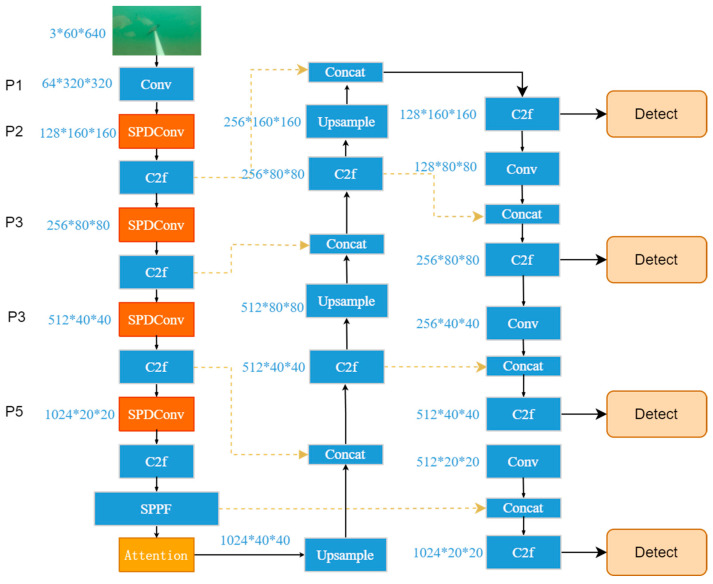
BSSFISH-YOLOv8 network structure.

Based on the original YOLOv8n model, adding a 160 × 160 small target detection layer can significantly improve the detection sensitivity of smaller targets. The specific method is to stack the fifth 80 × 80 size feature layer of the backbone and the upsampled feature layer of the neck part and generate a deep semantic feature layer containing small target feature information through C2f and further upsampling processing. Then, this deep feature layer will be combined with the shallow position feature layer of the third layer in the backbone, thereby enhancing the ability of the 160 × 160 scale fusion feature layer to express small target semantics and position information. This allows small target feature information to be transferred to the other three scale feature layers along the downsampling path through the head structure, thereby improving the feature fusion capability of the network and further improving the detection accuracy of small targets. By adding a small target detection layer and combining SPD-Conv and Biformer, a BSSFISH-YOLOv8 network model with improved performance is formed. The overall structure is shown in Figure 5.

## 4. Results

### 4.1. Experimental Dataset and Experimental Settings

The fish dataset utilized in this paper was created by merging two components. A portion of the data is derived from public and free fish data provided by the Moreton Bay Environmental Education Center [42]. Data were acquired from a 2017–2021 video from Moreton Bay, Australia, using a typical baited underwater video setup at a depth of 2–5 m with a GoPro camera captured in 1080p resolution. The video data were captured under natural lighting conditions in Moreton Bay, with water visibility limited to the measured Secchi depth. The Norwegian Institute of Nature Research collected data on freshwater fish from Høyegga and Myggbukta in Inlandet, Norway [43]. The data include various species present in the Norwegian freshwater environment and were collected under daylight conditions with natural lighting. Fish are classified by species. The annotators are computer science students at the Norwegian University of Science and Technology (NTNU), who collected the relevant video data for later annotation, and who were assisted by scientists from the Norwegian Institute for Nature Research (NINA). NINA is Norway’s leading applied ecological research institute with expertise in genetics, populations, species, ecosystems and landscapes in terrestrial, freshwater, and coastal marine environments. The annotators followed certain rules during the annotation process: when the head of the fish was present in the image, it was annotated, and when 50% of the fish’s body was out of the image, it was not annotated. In addition, to ensure the accuracy of the annotation process, only distinguishable genera and species were annotated. The source data comprise associated annotation files, which are extracted and converted into YOLO format. After screening the data from both sources, we selected specific fish images as the data source and created the dataset. To reduce the risk of overfitting, we deleted highly similar images in the original dataset. When selecting dataset images, we covered as many scene conditions as possible to ensure the robustness of the model to various real-world situations. The annotation information of all images was checked again to ensure accuracy and consistency. We also removed irrelevant or erroneous images to further improve the quality of the dataset. The complete dataset contains about 2000 photos. To ensure that the dataset’s distribution is consistent, it is randomly divided into a training set and a test set using random sampling in a 7:3 ratio. Each image correlates to a YOLO label file, which contains category and coordinate information.

**Figure 6:**
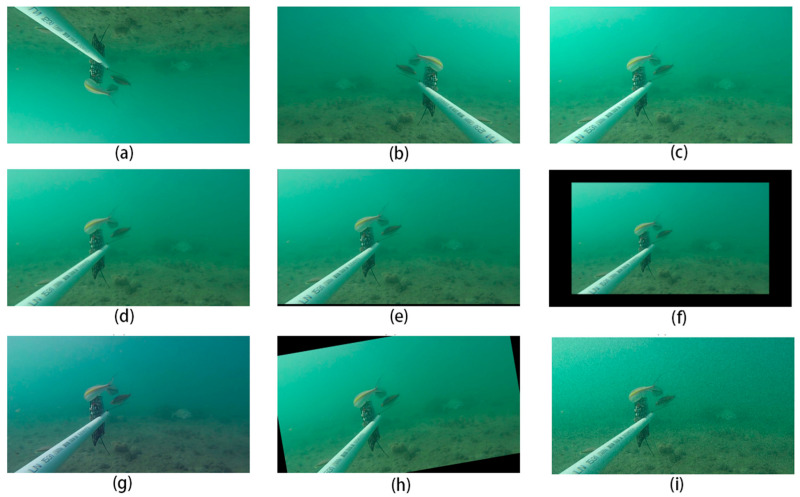
Examples of enhanced images: (**a**) vertical flip; (**b**) horizontal flip; (**c**) brightness adjustment; (**d**) Gaussian blur; (**e**) affine transformation translation; (**f**) affine transformation scaling; (**g**) channel addition; (**h**) rotate; (**i**) Gaussian noise.

The distribution of images in the dataset was analyzed to determine the next approach to data expansion: data augmentation enables network models to learn more extensive and generalized feature information, which improves model performance on unknown data, improves generalization, and reduces the risk of overfitting. In addition, data expansion can be used as a regularization method to help the model converge more consistently during training, especially when the model structure is complex or the training data are unevenly distributed. To increase the diversity and robustness of the experimental dataset, the following nine random combinations are employed to improve the training set in the picture dataset, and the annotation box is altered to adapt to the data-enhanced image. Finally, photographs with annotation boxes that are outside the image owing to image alteration are removed. Figure 6 depicts the enhanced images from each enhancing process. Table 1 shows how many samples are in the final dataset.

(a) Vertical flip: vertical flip with 50% probability.

(b) Horizontal flip: flips horizontally with 50% probability.

(c) Brightness adjustment: randomly scales the brightness of the image to between 80% and 120% of the original value.

(d) Gaussian blur: applies a Gaussian blur randomly in the range of 1–3.

(e) Affine transformation translation: randomly translates (−15, 15) pixels.

(f) Affine transformation scaling: randomly scales by a ratio of 0.8–0.9.

(g) Channel addition: randomly adds (−40, 40) pixel values to each channel.

(h) Rotate: randomly rotates the image from −15° to 15 °.

(i) Gaussian noise: adds Gaussian noise to the image; the scale of the noise is between 5 and 25.

**Figure 7:**
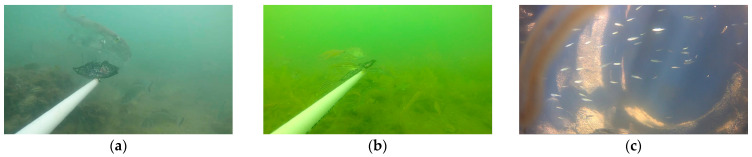
Typical images in the dataset: (**a**) blur; (**b**) occlusion; (**c**) small targets.

This dataset contains nine different types of fish. By analyzing the dataset, it can be found that there are problems in the dataset such as uneven distribution of samples, mutual occlusion of targets, turbid and unclear underwater images, and small targets. There are often differences in the numbers of different types of fish in natural waters. The number of samples of some fish may be large, whereas the number of samples of other fish is relatively small. As a result, the model training and recognition process may be more biased towards common fish, affecting the recognition effect of rare fish. Figure 7 visually displays representative images from the dataset. These images highlight the diversity and complexity of fish and underwater environments in real-life environments, highlighting the difficulties faced by underwater fish detection technology in natural waters. As shown in Figure 7a, due to factors such as water quality and insufficient light, underwater images often appear turbid, blurred, or scattered. In this case, the fish features in the image may not be visible enough, making it difficult for the model to accurately capture the details of the fish, thus affecting the fish recognition effect. The fish may block each other or be blocked by some obstacles, as shown in Figure 7b. This situation prevents the characteristics of some fish from being completely observed, which in turn affects the model’s accurate identification of this type of fish. As shown in Figure 7c, there are some smaller fish among the underwater fish, and these fish may appear as smaller targets in the image. Because the target is small, its feature information may be relatively limited, which brings challenges to the detection and recognition process of the model.

The experimental equipment is shown in Table 2 The experimental settings are set according to the officially recommended parameters of YOLOv8. The details are shown in Table 3.

### 4.2. Evaluation Indicators

To test the detection performance of the proposed improved model, precision, recall, mAP@50, mAP@50:95, the number of model parameters, and the model size are used as evaluation indicators. The following parameters are used in the formulas of some of the above evaluation indicators: TP (True Positive, predicted to be a positive sample and is a positive sample), FP (False Positive, predicted to be a positive sample but is a negative sample), and FN (False Negative, predicted as a negative sample but is a positive sample). These are key indicators for evaluating model performance. IoU (Intersection over Union) measures the ratio of the intersection and union of the predicted bounding box and the true box.

Precision is the ratio of the number of positive samples predicted by the model to the number of all detected samples. The calculation formula is as shown in Formula (8):

(8) $$P=\frac{TP}{TP+FP}$$

Recall is the ratio of the number of positive samples correctly predicted by the model to the number of positive samples that occurred. The recall rate is calculated as shown in Formula (9):

(9) $$R=\frac{TP}{TP+FN}$$

Average precision (AP) represents the area under the precision–recall curve, and the calculation formula is as shown in Formula (10):

(10) $$\mathrm{AP}=\int_{0}^{1}p(R)\mathrm{dr}$$

The mean average precision (mAP) is the result obtained by the weighted average of AP values for all sample categories. Its calculation formula is as shown in Formula (11):

(11) $$m\mathrm{AP}=\frac{\sum_{i=1}^{n}\mathrm{AP}\left(i\right)}{n}$$

AP(i) in Formula (11) represents the AP value with category index value i, and N represents the number of categories in this category. There are N samples in the training dataset (N is 9 in this article). mAP@50 represents the average accuracy when the IoU of the detection model is set to 50%, and mAP@50:95 represents the average accuracy when the IoU of the detection model is set to 50% to 95% (values at intervals of 5%).

Both mAP@50 and mAP@50:95 were used for evaluation on the mean average precision metric. Although mAP@50 can be used to quickly evaluate model performance, to evaluate the model more comprehensively and accurately, we also considered multiple IoU threshold ranges for mAP@50:95. This approach covers multiple thresholds from 50% to 95%, which can fully reflect the performance of the model under different accuracy requirements. Using a high IoU threshold can evaluate the model’s ability to accurately locate fish targets, and a low IoU threshold evaluates the model’s tolerance and ability to handle noise. This detailed evaluation helps to uncover differences in the performance of the model under various conditions, allowing us to more accurately understand the strengths and weaknesses of the model.

Flops refers to the number of floating point operations and is used to measure the computational complexity of the model. Params represent the total number of parameters in the model and are used to evaluate the size of the model.

### 4.3. Experiment Results

#### 4.3.1. Experimental Analysis of Introducing the SPD-Conv Model

To test the performance of SPD-Conv in detecting target fish with low definition and a small pixel ratio, the SPD-Conv module was introduced into the YOLOv8n network, and a comparative experiment was conducted with the original model. The other parts of the network remained unchanged. The data are recorded in Table 4. Through experimental analysis, the performance of the original YOLOv8n model and the improved YOLOv8n-SPD model after the introduction of the SPD-Conv module were compared. The performance of the two models was analyzed in detail for four key indicators: mAP@50, mAP@50:95, precision, and recall.

After introducing the SPD-Conv module, the YOLOv8n-SPD model achieved significant improvements in all indicators. Specifically, compared with the original YOLOv8n model, the YOLOv8n-SPD model improved the mAP@50 indicator by 0.5% to 87.1%, and the mAP@50:95 indicator increased by 0.6% to 56.4%. In addition, the precision and recall of the YOLOv8n-SPD model increased by 1.5% and 1.1%, reaching 87.8% and 80.1%, respectively. These results show that the introduction of the SPD-Conv module has a significant positive impact on the performance of the YOLOv8n model and can effectively improve the accuracy and recall rate of target detection, thus providing more reliable results for practical applications.

#### 4.3.2. Adding BiFormer Block

By adding the BiFormer attention mechanism to the YOLOv8n feature fusion module, the performance of the improved YOLOv8n-BiFormer model was compared with the original YOLOv8n model. The experimental results are shown in Table 5. The YOLOv8n-BiFormer model that introduces the BiFormer attention mechanism shows a higher accuracy and recall rate in target detection. For the mAP@50 indicator, the YOLOv8n-BiFormer model reached 87.6%, which was 1% higher than the YOLOv8n model. On the mAP@50:95 indicator, the YOLOv8n-BiFormer model reached 57.1%, an increase of 1.3% compared to the YOLOv8n model. In addition, the results of precision and recall show that the accuracy of the YOLOv8n-BiFormer model dropped slightly to 83.8%, whereas the accuracy of the YOLOv8n model was 86.3%. However, the recall rate of the YOLOv8n-BiFormer model is significantly improved, reaching 83.5%, whereas the recall rate of the YOLOv8n model is 79.0%.

These experimental results show that by introducing the BiFormer attention mechanism into the feature fusion module, the YOLOv8n-BiFormer model achieves better performance in underwater biological target detection. It can effectively solve the problems of missed detection and false detection caused by the complexity of the underwater environment and improve the accuracy and recall rate of target detection.

**Figure 8:**
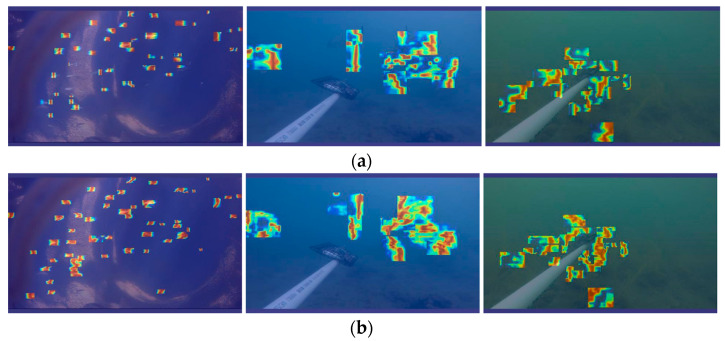
Comparison of heat maps: (**a**) YOLOv8n; (**b**) BSSFISH-YOLOv8.

To vividly demonstrate the effect of introducing the BiFormer attention mechanism, gradient-weighted class activation mapping (Grade CAM) was used to generate heat maps [44] of YOLOv8n and YOLOv8n-BiFormer, as shown in Figure 8. Heatmaps show the areas of the feature map that the model focuses on by backpropagating gradient values, with pixels with higher gradients shaded in dark red and pixels with lower gradients shaded in dark blue.

By observing the heat map results, the following conclusions can be drawn: YOLOv8n-BiFormer pays more attention to the detection area of the fish target body than YOLOv8n. It can be seen in the heat map that YOLOv8n-BiFormer produces thicker red shadows in the area around the fish target, indicating that the model pays more attention to extracting detection information at key positions of fish. Compared with YOLOv8n, YOLOv8n-BiFormer has a better suppression effect on background noise. In the heat map, YOLOv8n-BiFormer pays more limited attention to the background area and focuses more on the fish target itself, which helps reduce false detections caused by low definition. In addition, YOLOv8n-BiFormer pays more attention to small target individuals. A significant increase in attention to small-sized fish targets can be observed in the heat map, which makes the model perform more accurately when detecting small objects. After adding the attention mechanism, the network’s attention is more focused on the center point of the object. The network focuses more attention on the central region of the fish target, which helps improve the accuracy of predicted bounding boxes. Through heat map analysis, it can be seen that after the introduction of the BiFormer attention mechanism, the network has made significant improvements in focusing on fish targets, suppressing background noise, and improving the accuracy of predicted bounding boxes, thus effectively improving the accuracy of underwater fish object detection.

#### 4.3.3. Experimental Analysis of Importing Small Target Detection Layer (STDL)

The STDL can expand the receptive field, which allows the algorithm to detect and identify smaller fish more effectively. To verify whether the improved method can optimize the algorithm, the same experimental parameters are used to compare the detection effects before and after introducing the small target detection layer. According to the experimental results in Table 6, it can be seen that the effect of introducing the small target detection layer is more prominent. In terms of the mAP@50 indicator, YOLOv8n-STDL improved by 1.6% compared to YOLOv8n, reaching 88.2%. In terms of the mAP@50:95 indicator, YOLOv8n-STDL improved by 1.5% compared to YOLOv8n, reaching 57.3%. In terms of accuracy indicators, YOLOv8n-STDL and YOLOv8n perform similarly, 86.9% and 86.3%, respectively. In terms of recall rate, YOLOv8n-STDL increased by 2.6% compared to YOLOv8n to 81.6%, indicating that the model detected more targets. In summary, YOLOv8n has been successfully optimized by introducing the small target detection layer, achieving significant improvements in small target detection, improving the mAP index and recall rate, and effectively improving the accuracy and breadth of detection of small target fish.

**Figure 9:**
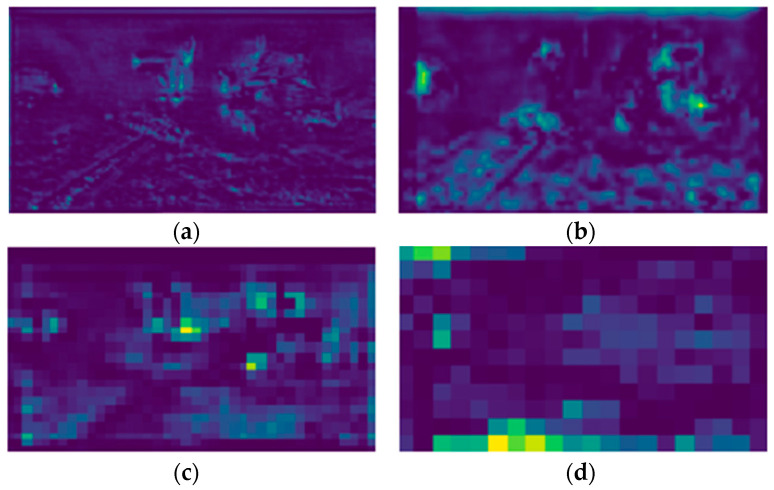
Feature maps of different scales: (**a**) 160 × 160; (**b**) 80 × 80; (**c**) 40 × 40; (**d**) 20 × 20.

Figure 9 shows examples of different output feature maps at four detection scales. It can be found that by adding a small target detection layer (STDL), more detailed information is retained on the 160 × 160 scale feature map, which facilitates the model in analyzing the image. The capture of details is more conducive to the detection of small targets.

### 4.4. Ablation Experiment

To evaluate the effectiveness of various improvement strategies proposed in this study, a series of ablation experiments was conducted on the baseline model YOLOv8n, and the dataset constructed by this study was used for testing. The experimental results are shown in Table 7 below, where the “√” mark indicates the adoption of the corresponding improvement strategy.

Experimental results show that each improvement strategy has different degrees of performance improvement when applied to the baseline model YOLOv8n. Specifically, after using SPD-Conv to replace some of the convolutional layers of the original backbone network, the two indicators mAP@50 and mAP@50:95 increased by 0.5% and 0.6%, respectively. We continue to introduce the BiFormer attention module into the backbone network. The efficient attention mechanism in BiFormer improves attention to key information in the feature map, making the two indicators mAP@50 and mAP@50:95 improve by 1.0% and 1.0%, respectively, compared with the baseline model. Moreover, the relatively simple structure of the BiFormer module adds only a small amount of model parameters and calculations after being added to the network. After finally adding the small target detection layer, the two indicators mAP@50 and mAP@50:95 reached 88.5% and 58.2%, respectively, which was 1.9% points and 2.4% higher than the baseline, respectively.

With the gradual introduction of these modules, the mAP@50 and mAP@50:95 indicators show a gradual improvement trend, indicating that the addition of these modules has a positive impact on the algorithm performance. Most of the detection indicators of the improved model have been effectively improved. It can also be observed that with the introduction of modules, the number of network parameters and the amount of calculations have increased. This may cause the model structure to become complex and the inference time to become longer.

### 4.5. Contrast Experiment

To further verify the superiority and effectiveness of the improved algorithm proposed in this section, comparative experiments were conducted using different types of target detection algorithms. YOLOv3 is the first algorithm to introduce multi-scale detection. This experiment uses its lightweight version YOLOv3-tiny. By adopting the idea of CSPNet [45] to build CSPDarknet53 as a new backbone network structure, YOLOv4 successfully reduces the amount of calculation while maintaining detection accuracy. YOLOv5 uses Mosaic data enhancement and Focus structure to improve the training speed and detection accuracy of the model and reduce the number of parameters and computational burden. YOLOv7 adopts the efficient ELAN [46] network architecture to further optimize target detection performance. SSD and Faster-RCNN are classic one-stage and two-stage target detection algorithms, respectively.

The comprehensive comparative experimental results are shown in Table 8. Experimental results show that the BSSFISH-YOLOv8 model exhibits outstanding performance compared to other models in multiple dimensions. Especially on the two key performance indicators of mAP@50 and mAP@50:95, BSSFISH-YOLOv8 achieved results of 88.5% and 58.2%, respectively, which not only significantly surpassed other YOLO series models but was also better than SSD and Faster-RCNN, two classic target detection algorithm models.

Furthermore, in terms of parameter efficiency, BSSFISH-YOLOv8 has 4.4 million parameters, compared to 8.7 million for YOLOv3-tiny and 25.3 million for SSD. At the same time, its calculation rate is only 15.1GFLOPs, significantly lower than SSD and Faster-RCNN. This demonstrates that BSSFISH-YOLOv8 makes efficient use of computer resources while providing better performance. Early YOLO series algorithms, such as YOLOv3, have complicated structures and a high number of parameters, yet their detection accuracy is quite low. Although YOLOv3-Tiny uses a lightweight model, it loses a significant amount of detection accuracy. In comparison, YOLOv5n and YOLOv8n have advanced with lower model sizes, fewer parameters, and improved detection performance. However, these two models cannot match the requirements for reliable fish detection in natural aquatic environments, and their detection accuracy is much lower than the better method given in this chapter. YOLOv7-tiny has issues in both model size and detecting performance. A comprehensive analysis of experimental findings reveals that BSSFISH-YOLOv8 has the highest average detection accuracy and overall detection performance.

### 4.6. Analysis of Improvement Effects

**Figure 10:**
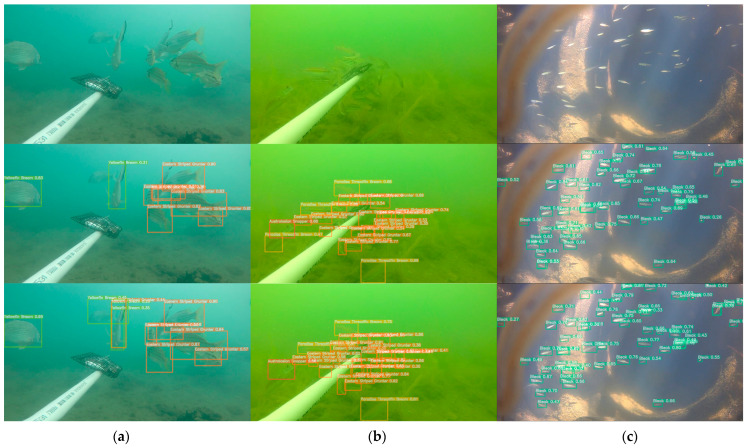
Comparison of improvement effects. From top to bottom: original images; YOLOv8n; BSSFISH-YOLOv8. (**a**) blur; (**b**) occlusion; (**c**) small targets.

This section uses three pictures of different scenes for effect testing to visually evaluate and demonstrate the detection effects of the original YOLOv8n and the improved BSSFISH-YOLOv8 model. In the experiment, the input image size was set to 640 × 640, and the confidence threshold was set to 0.25. The experimental results are shown in Figure 10. The three images have problems such as mutual occlusion, turbid and unclear underwater images, and small fish targets. By using the improved algorithm model, these problems have been effectively solved, and the detection effect has been significantly improved. The improved model can more accurately identify targets when dealing with mutual occlusion, such as in the first and second pictures, and it recognizes more fish than YOLOv8n. For example, when dealing with underwater blurred images in the second picture, it can improve the detection accuracy, and the confidence of most detected fish has been improved; when dealing with smaller targets, such as in the third picture, it can better capture the target and reduce the missed detection rate and false detection rate. Therefore, from the perspective of improvement effect, the improved model has shown obvious advantages in error detection, missed detection, and confidence improvement, effectively improving the detection effect on images.

**Figure 11:**
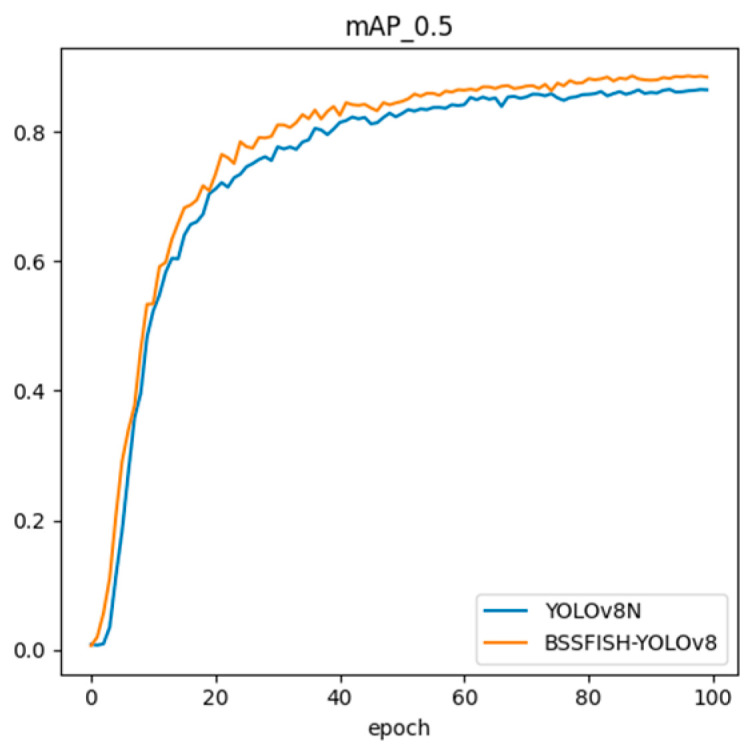
mAP@50 curve.

Figure 11 visually shows the changes in the mAP@50 indicator during training between YOLOv8n and the improved algorithm. The improved model can improve training indicators faster during training and achieve higher average detection accuracy when converging. Specifically, BSSFISH-YOLOv8 not only rapidly improves detection accuracy in the early stages of training but also maintains a higher mAP value in the later stages of training, which indicates that it is more accurate in detecting fish targets. During the entire training process, the curve of BSSFISH-YOLOv8 is always higher than the curve of YOLOv8n, indicating that it performs better than YOLOv8n in detection and recognition tasks.

**Figure 12:**
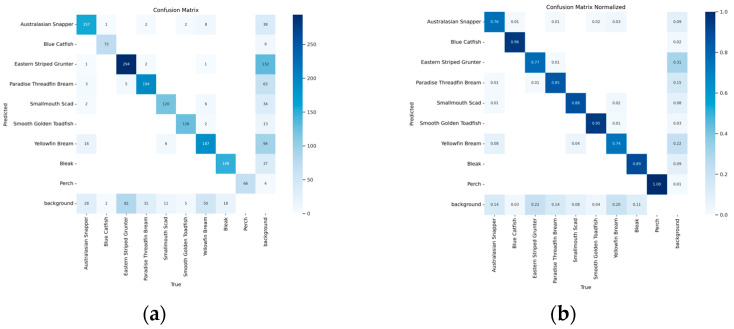
Confusion matrix for identification of different fish species. (**a**) quantitative information (**b**) ratio information.

**Figure 13:**
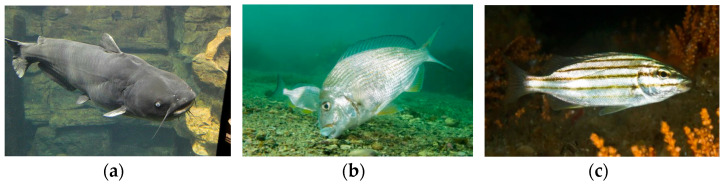
Demonstration of cases with higher and lower fish detection accuracy: (**a**) Blue Catfish; (**b**) Yellowfin Bream; (**c**) Eastern Striped Grunter.

As shown in Table 9, the BSSFISH-YOLOv8 model clearly outperforms the YOLOv8n model in detecting various fish species. In the Australasian Snapper category, BSSFISH-YOLOv8 had an accuracy of 85.9% on the mAP@50 indication, 3% greater than YOLOv8n’s 82.9%. On the more severe mAP@50:95 indication, BSSFISH-YOLOv8 did even better, attaining 60.1%. Compared to YOLOv8n’s 56.5%, it rose by 3.6%. This huge improvement shows that BSSFISH-YOLOv8 can better recognize Australasian Snapper. BSSFISH-YOLOv8 also excels in data performance for Eastern Striped Grunter. On mAP@50, BSSFISH-YOLOv8 is 2.7% higher than YOLOv8n, and on mAP@50:95, the advantage is even more pronounced, at 4.8% higher. This means that BSSFISH-YOLOv8 is more precise when processing the details and boundaries of fish like the Eastern Striped Grunter. For Smallmouth Scad, the mAP@50 of BSSFISH-YOLOv8 was 90.4%, 2.9% higher than the 87.5% of YOLOv8n. On mAP@50:95, this improvement persists, and BSSFISH-YOLOv8 outperforms YOLOv8n by 2.9%. These data demonstrate once more the superiority of BSSFISH-YOLOv8 when dealing with fish with complicated textures or shapes. The Smooth Golden Toadfish produced better results as well. BSSFISH-YOLOv8 has a modest advantage in mAP@50, but the advantage is more pronounced in mAP@50:95, increasing by 1.6%. This demonstrates that BSSFISH-YOLOv8 can better grasp details while dealing with the high-precision requirements of this type of fish. For Perch fish, although the two models are tied on mAP@50, both reaching 99.5%, BSSFISH-YOLOv8 leads by 1.6% on mAP@50:95, demonstrating that even at extremely high accuracy requirements, BSSFISH-YOLOv8 can maintain superior performance. Additionally, Figure 12 illustrates the confusion matrix of the recognition results between the different fish species on the test datasets. Figure 12a,b show the proportions and the quantitative information, respectively. Individual fish with higher (Blue Catfish) and lower (Yellowfin Bream, Eastern Striped Grunter) recognition accuracies are shown in comparison in Figure 13.

## 5. Conclusions

To effectively detect fish targets in natural water, this work presents an optimized structure known as BSSFISH-YOLOv8. The SPD-Conv module, which is based on the fundamental structure of the YOLOv8n model, retains discriminative feature information, effectively tackling the problem of low clarity in the underwater environment. Furthermore, BiFormer, which includes a dynamic sparse attention mechanism, considerably decreases the difficulties of missed and erroneous detection caused by underwater biological aggregation and mutual occlusion. To boost the network’s sensitivity to small targets in complicated surroundings, the model’s receptive field and feature fusion mode were changed, resulting in a considerable improvement. Compared to YOLOv8n, the enhanced model’s mAP@50 and mAP@50:95 increased by 1.9% and 2.4%, respectively. In addition, it outperforms some traditional target detection algorithms in terms of detection accuracy.

At present, there are still some problems in conducting fishery resource surveys through target detection technology, such as the same fish being counted multiple times, affecting the accuracy of the results. To solve this problem, cooperation with ichthyologists can be considered in the future. Since water bait is used to drive fish to the underwater camera to obtain data, by analyzing the regularity of fish feeding behavior, their activity patterns under specific time and environmental conditions can be found. By cooperating with ichthyologists, in-depth research on the feeding behavior of fish in different environments is established, including their feeding frequency, time interval, group behavior, and individual characteristics during feeding, as is a detailed fish behavior model. Using this model, the repeated detection coefficient in feeding behavior is calculated, which can reflect the probability of the same fish being detected multiple times during feeding. Then, this coefficient is applied to the target detection results to correct the number of fish detected and eliminate the error caused by repeated detection. Ultimately, this method can obtain a more accurate and approximate estimate of the number of fish, thereby improving the accuracy and reliability of fishery resource surveys.

The advanced target detection technology is not only suitable for underwater species monitoring but also has broad application prospects in fish population monitoring in intensive farming systems. In intensive breeding systems, target detection technology can monitor the growth, health status, and behavioral patterns of fish in real time, helping farmers optimize feeding strategies, detect and deal with diseases in a timely manner, reduce losses, and improve overall breeding efficiency and production. For example, by analyzing video data in real time, abnormal fish behavior or health problems can be quickly discovered, and corresponding measures can be taken, such as adjusting feed formulas or administering medication. With the development of edge computing and hardware acceleration technology, target detection technology will be significantly improved in terms of real-time performance and efficiency. These technologies make it possible to deploy systems on site at farms that can instantly process and analyze large amounts of video data, providing real-time monitoring results and decision support. The application of target detection technology will greatly improve the level of fish monitoring and management, promote the development of the aquaculture industry in the direction of intelligence and automation, and improve production efficiency and sustainability.

## Figures and Tables

**Table 1:** Number of samples in the dataset.

Fish Categories	Total Number	Class Distribution of the Test Dataset
Australasian Snapper	1389	207
Blue Catfish	450	76
Eastern Striped Grunter	2023	381
Paradise Threadfin Bream	1605	229
Smallmouth Scad	1144	137
Smooth Golden Toadfish	947	133
Yellowfin Bream	1806	254
Bleak	1592	166
Perch	458	66

**Table 2:** Server hardware environment.

Configuration	Parameter
CPU	Intel Xeon Platinum 8255 CPU
GPU	NVIDIA GeForce RTX 2080Ti GPU
Operating system	Ubuntu
Deep learning framework	Pytorch 2.0
Programming language	Python3.9
CUDA	11.8

**Table 3:** Experimental settings.

Parameter Name	Set-Up
image size	640 × 640
batch size	8
learning rate	0.01
optimizer	SGD
momentum factor	0.937
epochs	100

**Table 4:** Experimental analysis of introducing the SPD-Conv module.

Model	mAP@50 (%)	mAP@50:95 (%)	Precision (%)	Recall (%)
YOLOv8n	86.6	55.8	86.3	79.0
YOLOv8n-SPD	87.1	56.4	87.8	80.1

**Table 5:** Experimental analysis of adding the BiFormer module.

Model	mAP@50 (%)	mAP@50:95 (%)	Precision (%)	Recall (%)
YOLOv8n	86.6	55.8	86.3	79.0
YOLOv8n-BiFormer	87.6	57.1	83.8	83.5

**Table 6:** Experimental analysis of introducing a small target detection layer (STDL).

Model	mAP@50 (%)	mAP@50:95 (%)	Precision (%)	Recall (%)
YOLOv8n	86.6	55.8	86.3	79.0
YOLOv8n-STDL	88.2	57.3	86.9	81.6

**Table 7:** Experiment results of the ablation experiment.

SPD-Conv	Biformer	STDL	mAP@50 (%)	mAP@50:95 (%)	Params (M)	GFlops (G)
			86.6	55.8	3.0	8.2
√			87.1	56.4	4.2	11.0
√	√		87.6	56.8	4.4	11.0
√	√	√	88.5	58.2	4.4	15.1

**Table 8:** Experiment results of contrast experiment.

Model	mAP@50 (%)	mAP@50:95 (%)	Params (M)	GFlops (G)
BSSFISH-YOLOv8	88.5	58.2	4.4	15.1
YOLOv8n	86.6	55.8	3.0	8.2
YOLOv7-tiny	84.7	50.0	6.0	13.2
YOLOv5n	81.4	42.2	1.8	4.3
YOLOv3-tiny	82.8	47.1	8.7	12.9
SSD	73.5	42.6	25.3	116.2
Faster-RCNN	80.9	44.4	133.8	368.3

**Table 9:** Comparison of detection accuracy.

Fish Categories	BSSFISH-YOLOv8 -mAP@50 (%)	BSSFISH-YOLOv8 -mAP@50:95 (%)	YOLOv8n -mAP@50 (%)	YOLOv8n -mAP@50:95 (%)
Australasian Snapper	85.9	60.1	82.9	56.5
Blue Catfish	98.0	59.6	97.7	64.2
Eastern Striped Grunter	76.9	39.1	74.2	34.3
Paradise Threadfin Bream	85.6	53.4	82.4	48.8
Smallmouth Scad	90.4	62.0	87.5	59.1
Smooth Golden Toadfish	97.2	71.4	96.9	69.8
Yellowfin Bream	74.0	49.9	73.6	46.2
Bleak	88.6	43.5	84.6	39.6
Perch	99.5	85.4	99.5	83.8
Total	88.5	58.2	86.6	55.8

## Data Availability

The code can be requested from the corresponding authors. The data are from public datasets, which are introduced in Section 4.1.
